# The prognostic value of the neutrophil-to-lymphocyte ratio, platelet-to-lymphocyte ratio, and prognostic nutritional index for survival in patients with colorectal cancer

**DOI:** 10.1515/med-2025-1214

**Published:** 2025-06-11

**Authors:** Xianya Zhu, Qiuping Xu, Yi Zhou, Chunrong Zhu, Lili Zeng

**Affiliations:** Department of Hematology and Oncology, Suzhou Wuzhong People’s Hospital, Suzhou, 215000, China; Department of Oncology, The First Affiliated Hospital of Soochow University, 188 Shizi Street, Canglang District, Suzhou, Jiangsu, 215000, China

**Keywords:** inflammatory, nutrition, biomarker, colorectal cancer, prognosis

## Abstract

To analyze the impact of the neutrophil-to-lymphocyte ratio (NLR), platelet-to-lymphocyte ratio (PLR), prognostic nutritional index (PNI), and body mass index on the prognosis of colorectal cancer patients and to assess their clinical value. This cohort study included patients who underwent radical resection (R0 resection) for colorectal cancer. Peripheral blood laboratory test results for all patients were obtained within 1 week prior to surgery for the calculation of the NLR, PLR, and PNI. A total of 201 patients were included in the analysis. A total of 19 patients experienced relapse, and 30 patients died. The median follow-up duration was 39.2 months. Receiver operating characteristic curve analysis indicated that the PNI demonstrated moderately high predictive accuracy for overall survival (OS), with an area under the curve of 67.31%. After stratification based on the cutoff value, patients with a PNI of ≥44.48 exhibited a better survival prognosis than those with a PNI of <44.48 (*P* = 0.001). However, according to the univariate Cox regression analysis, the PNI was significantly associated with OS (hazard ratio: 0.29; 95% confidence interval: 0.14–0.62; *P* = 0.001). The PNI, rather than the NLR or PLR, exhibited better predictive performance. After classifying patients based on the PNI cutoff value, patients with a high PNI had better survival outcomes.

## 1 Introduction

Colorectal cancer is the third most common malignant tumor in the world and a leading cause of cancer-related mortality. As economic levels improve and dietary habits and structures evolve, the incidence of colorectal cancer is increasing annually, with a concurrent decrease in the average age at diagnosis. The survival rate, however, has not improved significantly [1,2,3].

At present, patients with colorectal cancer are predominantly treated through a combination of radical surgical resection, radiotherapy, and chemotherapy. When colorectal cancer progresses to distant metastasis, the 5-year survival rate decreases from 90% in the early stages to 15% [4,5,6]. Identifying an efficient prognostic biomarker for colorectal cancer is crucial for early detection as well as for assessing treatment efficacy and prognostic outcomes. At present, carcinoembryonic antigen (CEA) is widely utilized as a clinical tumor marker for detecting the presence and progression of tumors. However, this approach has limitations and does not account for the equilibrium between protumorigenic immunity and the host’s antitumor defenses [7,8]. Studies have demonstrated that tumor-associated inflammatory cells can influence tumor biology both directly and indirectly, contributing to extracellular matrix remodeling and angiogenesis and closely associated with tumorigenesis and metastasis [9,10,11].

The platelet-to-lymphocyte count ratio (PLR) and the neutrophil-to-lymphocyte count ratio (NLR) can effectively reflect the systemic inflammatory status and can be utilized to assess the dynamic balance between tumor-promoting immunity and the body’s antitumor defenses [12,13,14]. The prognostic nutritional index (PNI) is an indicator that reflects the immune-nutritional status of the body [15,16]. In recent years, numerous scholars have investigated the associations between body mass index (BMI) and surgical outcomes, including safety, postoperative complications, and survival rates, in patients with colorectal cancer [17,18,19]. Studies have also demonstrated that these indicators are closely associated with patients’ systemic immune status, distant metastasis of tumors, and survival outcomes [20,21]. This study investigated the influence of several promising indicators and examined how inflammation-related markers affect the survival outcomes of colorectal cancer patients by jointly analyzing multiple metrics. This approach aimed to identify more precise prognostic factors to support evidence-based clinical decision-making.

## 2 Methods

### 2.1 Participants

We collected medical records from patients who underwent radical resection for colorectal cancer. The data were obtained from Suzhou Wuzhong People’s Hospital and the First Affiliated Hospital of Soochow University between June 2020 and June 2021. A follow-up period of 3 years was used for all patients.

### 2.2 Selection criteria

The inclusion criteria were as follows: (1) diagnosed with colorectal cancer through imaging and pathological examination; (2) underwent radical resection of colorectal cancer (R0 resection); (3) patients and their families provided informed consent; and (4) provided complete and detailed medical records.

The exclusion criteria were as follows: (1) patients who received chemoradiotherapy, targeted therapy, or hormone therapy prior to surgery; (2) patients who had heart, liver, lung, or renal insufficiency; (3) patients who had active infection or rheumatic immune disease; (4) patients who had other malignant tumors; (5) patients who received parenteral nutrition within 2 weeks preceding surgery; and (6) patients who required emergency surgery due to conditions such as bleeding, perforation, or intestinal obstruction.

### 2.3 Data collection

The patients’ clinical and pathological characteristics, including age, sex, height, body weight, BMI, tumor location, differentiation grade, and TNM stage, were meticulously documented at admission. BMI was calculated as body mass in kilograms divided by the square of height in meters (BMI = kg/m^2^). Clinical standards categorize BMI into three groups: less than 18.5 kg/m^2^ (underweight), 18.5–24.9 kg/m^2^ (normal weight), and 25 kg/m^2^ or greater (overweight/obesity). Peripheral blood, including neutrophil, lymphocyte, and platelet counts and serum albumin measurements, was collected from all patients within 1 week prior to surgery. The following formulas were applied to calculate additional indices: NLR, the absolute neutrophil count divided by the absolute lymphocyte count; PLR, the absolute platelet count divided by the lymphocyte count; and PNI, the serum ALB level plus five times the total lymphocyte count. Postoperative follow-up was conducted for patients at intervals of every 3 months during the initial 2 years and every 6 months in the subsequent year. The primary endpoint for follow-up observation was overall survival (OS), with the follow-up period ending in May 2024 or upon patient death.

### 2.4 Statistical analysis

Data analysis was performed using the R program (version 4.3.2). The NLR, PLR, and PNI were categorized into high and low groups based on the optimal cutoff values derived from receiver operating characteristic (ROC) curve analysis. ROC curve analysis was conducted to evaluate the diagnostic value of each detection index by comparing the sensitivity, specificity, accuracy, optimal cutoff value, and area under the curve (AUC) against the prognostic outcome. The chi-square test or Fisher’s exact test was utilized to assess the correlation between count data and patients’ clinical pathology. The Kaplan‒Meier method was used to plot survival curves and estimate survival rates, and the log-rank test was used to compare survival outcomes. The Cox regression model was applied for the analysis of prognostic factors, encompassing both univariate and multivariate approaches. A *P*-value of less than 0.05 was considered to indicate statistical significance.


**Ethical approval:** This research was performed in accordance with the Declaration of Helsinki and was approved by the Ethics Committee of Suzhou Wuzhong People’s Hospital and the First Affiliated Hospital of Soochow University.
**Informed consent:** All patients provided informed consent.

## 3 Results

### 3.1 Participant characteristics

This study included a total of 212 patients who were diagnosed with colorectal cancer; 11 patients were lost to follow-up, for a loss-to-follow-up rate of 5.2%. There were 55 participants with colon cancer, 143 with rectal cancer, and 3 with unspecified colorectal cancer locations. Among them, 82 were women, accounting for 40.8%, and 119 were men, accounting for 59.2%. The age of the patients ranged from 29 to 98 years, with a median age of 64.6 years (Q1, Q3: 57–74). During the follow-up period, 19 patients experienced disease relapse, and 30 patients passed away. The median follow-up duration was 39.2 months, as detailed in Table 1.

**Table 1:** Clinicopathological characteristics of colorectal cancer patients at follow-up

Variable	Median (Q1–Q3)/frequency
*N*	201
Age	66 (57–74)
BMI	22.84 (20.7–25.11)
PNI	45.85 (42.45–48.65)
NLR	2.3 (1.75–3.51)
PLR	155.67 (111.22–205.36)
Follow-up (month)	39.2 (35.033–41.267)
**Sex**	
Male	119
Female	82
**Differentiation**	
Poorly	7
Moderately poor	44
Moderately	134
Moderately well	12
Well	4
**T stage**	
0/is	5
1	18
2	23
3	143
4	12
**N stage**	
0	116
1	51
2	34
**M stage**	
0	200
1	1
**Type of tumor**	
Colon	55
Rectum	143
Colorectal	3
**Chemotherapy**	
No	58
Yes	143
**Radiotherapy**	
No	164
Yes	37
**Outcome**	
Progress	19
Death	30
Steady/improvement	152
**Disease-free survival**	
No	49
Yes	152
**Overall survival**	
No	30
Yes	171

Note: Medians (Q1–Q3) or frequencies were calculated.

Abbreviations: BMI: body mass index; NLR: neutrophil-to-lymphocyte ratio; PLR: platelet-to-lymphocyte ratio; PNI: prognostic nutritional index.

### 3.2 Correlations between the NLR, PLR, and PNI and patient survival outcomes

**Figure 1:**
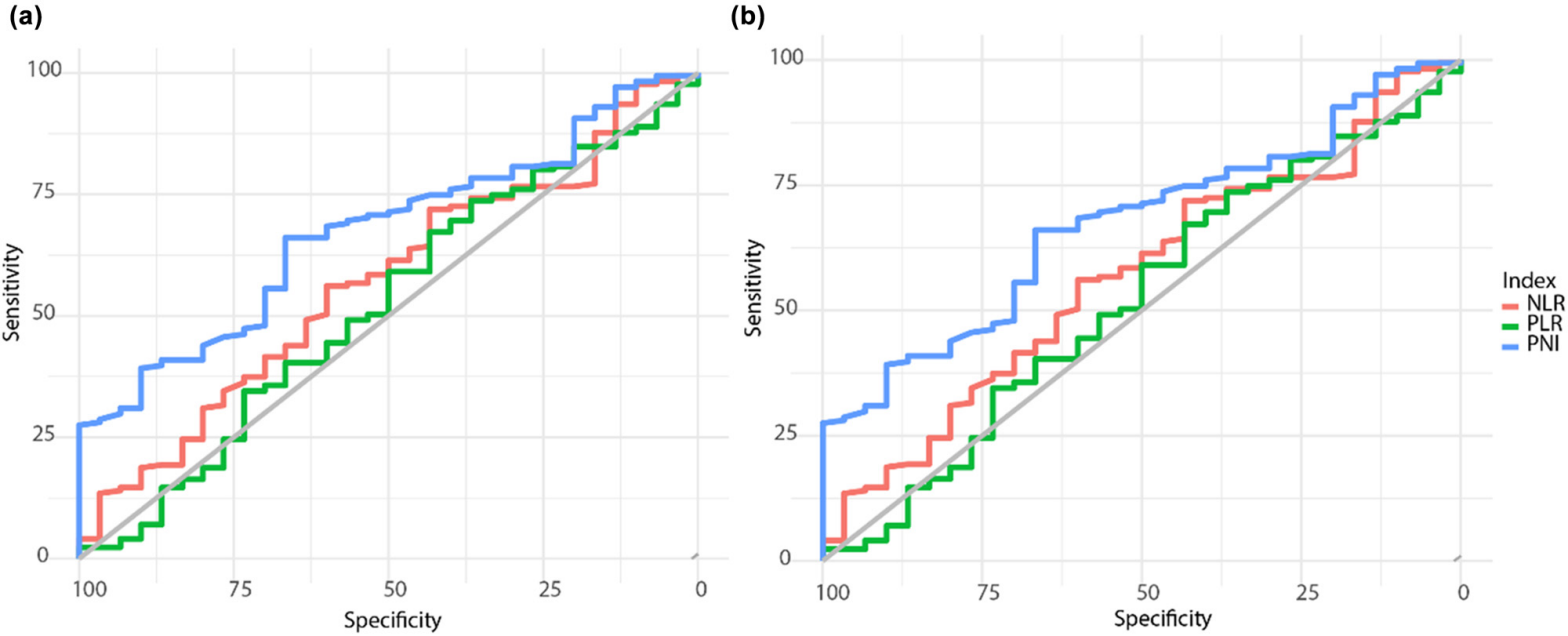
ROC curves showing the ability of the NLR, PLR, and PNI to predict OS and DFS in patients with colorectal cancer. (a) Predict OS results; (b) predict DFS results.

Using OS as the endpoint, ROC curve analysis was applied to determine the cutoff values for the NLR, PLR, and PNI, which were 2.45, 180.69, and 44.48, respectively (Figure 1a). The AUC, along with the sensitivity and specificity, for each of the three indices was as follows: for NLR, AUC = 56.92%, sensitivity = 56.14%, and specificity = 60%; for PLR, AUC = 52.09%, sensitivity = 67.25%, and specificity = 43.33%; and for PNI, AUC = 67.31%, sensitivity = 66.08%, and specificity = 66.67% (Table 2). The ROC curve for disease-free survival (DFS) is also shown in Figure 1b and Table 2.

**Table 2:** The ability of the NLR, PLR, and PNI to predict survival outcomes in patients with colorectal cancer

Outcomes	Type of patients	Index	Cutoff	AUC (%)	Sensitivity (%)	Specificity (%)	Accuracy (%)	AUC 95% CI (DeLong)	TN	TP	FN	FP
OS	Colorectal cancer	NLR	2.45	56.92	56.14	60	56.72	46.12–67.89%	18	96	75	12
		PLR	180.69	52.09	67.25	43.33	63.68	40.36–63.69%	13	115	56	17
		PNI	44.48	67.31	66.08	66.67	66.17	57.67–76.18%	20	113	58	10
	Colon cancer	NLR	3.57	65.34	66.67	65.22	65.45	43.27–87.41%	30	6	3	16
		PLR	141.57	52.90	88.89	32.61	41.82	31.35–74.45%	15	8	1	31
		PNI	37.03	71.98	44.44	91.30	83.64	53.74–90.22%	42	4	5	4
	Rectal cancer	NLR	2.45	52.74	55.00	57.72	57.34	39.88–65.61%	71	11	9	52
		PLR	99.42	52.07	30.00	81.30	74.13	37.46–66.68%	100	6	14	23
		PNI	44.48	64.98	65.00	70.73	69.93	54.02–75.94%	87	13	7	36
DFS	Colorectal cancer	NLR	1.97	56.59	39.47	75.51	48.26	47.75–65.43%	37	60	92	12
		PLR	143.31	54.52	59.21	55.1	58.21	45.25–63.8%	27	90	62	22
		PNI	49.45	62.92	28.29	100	45.77	54.98–70.85%	49	43	109	0
	Colon cancer	NLR	3.20	63.95	62.79	66.67	63.64	44.93–82.98%	8	27	16	4
		PLR	187.74	50.97	48.84	66.67	52.73	32.69–69.24%	8	21	22	4
		PNI	46.65	71.12	48.84	91.67	58.18	55.91–86.34%	11	21	22	1
	Rectal cancer	NLR	1.65	54.22	28.97	86.11	43.36	43.93–64.51%	31	31	76	5
		PLR	142.99	57.93	56.07	66.67	58.74	46.99–68.87%	24	60	47	12
		PNI	49.45	59.90	28.97	100	46.85	50.48–69.33%	36	31	76	0

Abbreviations: AUC: area under curve; DFS: disease-free survival; FN: false negative; FP: false positive; NLR: neutrophil-to-lymphocyte ratio; OS: overall survival; PLR: platelet-to-lymphocyte ratio; PNI: prognostic nutritional index; TN: true negative; TP: true positive.

### 3.3 Correlations between the NLR, PLR, PNI, BMI, and patients’ clinical and pathological characteristics

Based on the cutoff values for the NLR, PLR, and PNI, patients were categorized into a high NLR group (≥2.45), a low NLR group (<2.45), a high PLR group (≥108.69), a low PLR group (<108.69), a high PNI group (≥44.48), and a low PNI group (<44.48). The NLR was not significantly correlated with patient characteristics, except for receipt of radiotherapy (Table 3). The PLR was found to be correlated with patient tumor type and follow-up duration (Table 4). The PNI was associated with patient age, BMI, tumor type, receipt of chemotherapy, radiotherapy, and survival outcomes (Table 5). The BMI was associated with PNI (Table 6).

**Table 3:** Correlation between NLR categorization and patients’ clinical characteristics

Variable	NLR < 2.45	NLR ≥ 2.45	Statistic	*P* value
*N*	108	93		
Age	66 (57–70.25)	67 (58–77)	2.73	0.098
BMI	23.34 (20.758–25.477)	22.26 (20.62–24.48)	2.20	0.138
PNI	47.35 (45.112–50.35)	43.2 (39.85–46.1)	41.01	0.000
NLR	1.79 (1.435–2.092)	3.63 (2.83–4.88)	149.18	0.000
PLR	116.845 (86.243–158.52)	191.43 (157.86–230.95)	59.21	0.000
Follow-up (months)	39.867 (36.492–41.45)	38.733 (34.6–41.067)	1.97	0.161
**Sex**				
Male	62	57		
Female	46	36	0.31	0.678
**Differentiation**				
Poorly	3	4		
Moderately poor	29	15		
Moderately	69	65		
Moderately well	4	8		
Well	3	1	5.96	0.204
**T stage**				
0/is	2	3		
1	9	9		
2	14	9		
3	76	67		
4	7	5	1.07	0.905
**N stage**				
0	58	58		
1	30	21		
2	20	14	1.54	0.469
**M stage**				
0	107	93		
1	1	0	0.87	1.000
**Type of tumor**				
Colon	27	28		
Rectum	80	63		
Colorectal	1	2	1.26	0.528
**Chemotherapy**				
No	27	31		
Yes	81	62	1.69	0.218
**Radiotherapy**				
No	82	82		
Yes	26	11	4.99	0.033
**Outcome**				
Progress	10	9		
Death	12	18		
Steady/improvement	86	66	2.78	0.249
**Disease-free survival**				
No	22	27		
Yes	86	66	2.03	0.192
**Overall survival**				
No	12	18		
Yes	96	75	2.67	0.126

Note: Medians (Q1–Q3) or frequencies were calculated.

Abbreviations: BMI: body mass index; NLR: neutrophil-to-lymphocyte ratio; PLR: platelet-to-lymphocyte ratio; PNI: prognostic nutritional index.

**Table 4:** Correlation between PLR categorization and patients’ clinical characteristics

Variable	PLR < 108.69	PLR ≥ 108.69	Statistic	*P* value
*N*	132	69		
Age	67 (58–72)	66 (53–76)	0.496	0.481
BMI	23.21 (20.758–25.332)	22.48 (20.58–24.54)	1.660	0.198
PNI	46.55 (44.425–49.938)	42.75 (39.3–46.55)	27.038	0.000
NLR	2.04 (1.55–2.643)	3.63 (2.55–4.88)	42.948	0.000
PLR	124.155 (96.345–153.037)	223.48 (205.13–278.9)	135.268	0.000
Follow-up (months)	39.817 (37.075–41.633)	38.067 (32.433–40.6)	5.868	0.015
**Sex**				
Male	83	36		
Female	49	33	2.150	0.188
**Differentiation**				
Poorly	3	4		
Moderately poor	26	18		
Moderately	93	41		
Moderately well	7	5		
Well	3	1	3.730	0.452
**T stage**				
0/is	4	1		
1	10	8		
2	16	7		
3	95	48		
4	7	5	1.751	0.785
**N stage**				
0	75	41		
1	36	15		
2	21	13	0.830	0.683
**M stage**				
0	132	68		
1	0	1	1.923	0.350
**Type of tumor**				
Colon	27	28		
Rectum	104	39		
Colorectal	1	2	11.257	0.002
**Chemotherapy**				
No	40	18		
Yes	92	51	0.392	0.614
**Radiotherapy**				
No	104	60		
Yes	28	9	2.01	0.183
**Outcome**				
Progress	15	4		
Death	17	13		
Steady/improvement	100	52	2.565	0.274
**Disease-free survival**				
No	32	17		
Yes	100	52	0.004	1.000
**Overall survival**				
No	17	13		
Yes	115	56	1.268	0.308

Note: Medians (Q1–Q3) or frequencies were calculated.

Abbreviations: BMI: body mass index; NLR: neutrophil-to-lymphocyte ratio; PLR: platelet-to-lymphocyte ratio; PNI: prognostic nutritional index.

**Table 5:** Correlation between PNI categorization and patients’ clinical characteristics

Variable	PNI < 44.475	PNI ≥ 44.475	Statistic	*P* value
*N*	78	123		
Age	71 (63.25–78)	64 (56–69)	19.40	0.000
BMI	21.35 (19.86–24.188)	23.34 (21.415–25.88)	12.04	0.001
PNI	41.625 (39.15–42.9)	48.05 (46.225–51.025)	142.49	0.000
NLR	3.095 (2.34–4.797)	1.98 (1.54–2.705)	37.87	0.000
PLR	193.19 (147.765–231.122)	134.43 (97.435–171.41)	33.27	0.000
Follow-up (months)	39.083 (35.1–41.008)	39.5 (35.083–41.6)	0.97	0.325
**Sex**				
Male	47	72		
Female	31	51	0.06	0.887
**Differentiation**				
Poorly	4	3		
Moderately poor	16	28		
Moderately	52	82		
Moderately well	5	7		
Well	1	3	1.46	0.836
**T stage**				
0/is	3	2		
1	5	13		
2	13	10		
3	52	91		
4	5	7	5.31	0.269
**N stage**				
0	45	71		
1	19	32		
2	14	20	0.13	0.943
**M stage**				
0	78	122		
1		1	0.64	1.000
**Type of tumor**				
Colon	26	29		
Rectum	49	94		
Colorectal	3	0	7.63	0.019
**Chemotherapy**				
No	32	26		
Yes	46	97	9.20	0.003
**Radiotherapy**				
No	70	94		
Yes	8	29	5.64	0.028
**Outcome**				
Progress	6	13		
Death	20	10		
Steady/improvement	52	100	11.58	0.004
**Disease-free survival**				
No	26	23		
Yes	52	100	5.54	0.028
**Overall survival**				
No	20	10		
Yes	58	113	11.53	0.002

Note: Medians (Q1–Q3) or frequencies were calculated.

Abbreviations: BMI: body mass index; NLR: neutrophil-to-lymphocyte ratio; PLR: platelet-to-lymphocyte ratio; PNI: prognostic nutritional index.

**Table 6:** Correlation between BMI categorization and patients’ clinical characteristics

Variable	<18.5 kg/m^2^	18.5–24.9 kg/m^2^	≥25 kg/m^2^	Statistic	*P* value
*N*	15	134	52		
Age	71 (60.5–80)	67 (58–74.75)	64 (56.5–70)	4.47	0.107
BMI	17.3 (16.73–17.605)	22.125 (20.59–23.41)	27.005 (26.003–28.685)	137.20	0.000
PNI	42.65 (39.75–46.075)	45.425 (42.162–48.125)	47.775 (44.775–52.563)	16.47	0.000
NLR	2.21 (1.985–2.68)	2.46 (1.68–3.745)	2.19 (1.735–2.865)	1.41	0.494
PLR	174.32 (111.335–202.705)	158.075 (114.193–209.245)	140.105 (83.458–186.705)	4.47	0.107
Follow-up (month)	38.733 (34.117–41.783)	39.133 (34.675–41.033)	39.883 (37.333–42.342)	2.23	0.328
**Sex**	*n*				
Male	10	80	29		
Female	5	54	23	0.61	0.760
**Differentiation**					
Poorly		6	1		
Moderately poor	4	32	8		
Moderately	10	87	37		
Moderately well	1	7	4		
Well		2	2	4.65	0.808
**T stage**					
0/is	1	3	1		
1	2	12	4		
2	1	17	5		
3	9	95	39		
4	2	7	3	4.06	0.860
**N stage**					
0	7	76	33		
1	6	33	12		
2	2	25	7	2.76	0.591
**M stage**					
0	15	134	51		
1		38	1	2.88	0.330
**Type of tumor**					
Colon	5	94	12		
Rectum	10	2	39		
Colorectal			1	1.05	0.894
**Chemotherapy**					
No	6	41	11		
Yes	9	93	41		
**Radiotherapy**					
No	12	111	41		
Yes	3	23	11		0.76*
Outcome	1	13	5	2.61	0.283
**Progress**					
Death	1	25	4		
Steady/improvement	13	96	43	4.76	0.299
**Disease-free survival**					
No	13	96	43		
Yes	2	38	9	3.55	0.181
**Overall survival**					
No	14	109	48		
Yes	1	25	4	4.42	0.118

Note: Medians (Q1–Q3) or frequencies were calculated. *: Fisher’s exact test.

Abbreviations: BMI: body mass index; NLR: neutrophil-to-lymphocyte ratio; PLR: platelet-to-lymphocyte ratio; PNI: prognostic nutritional index.

### 3.4 Relationships between the NLR, PLR, PNI, BMI, and OS

**Figure 2:**
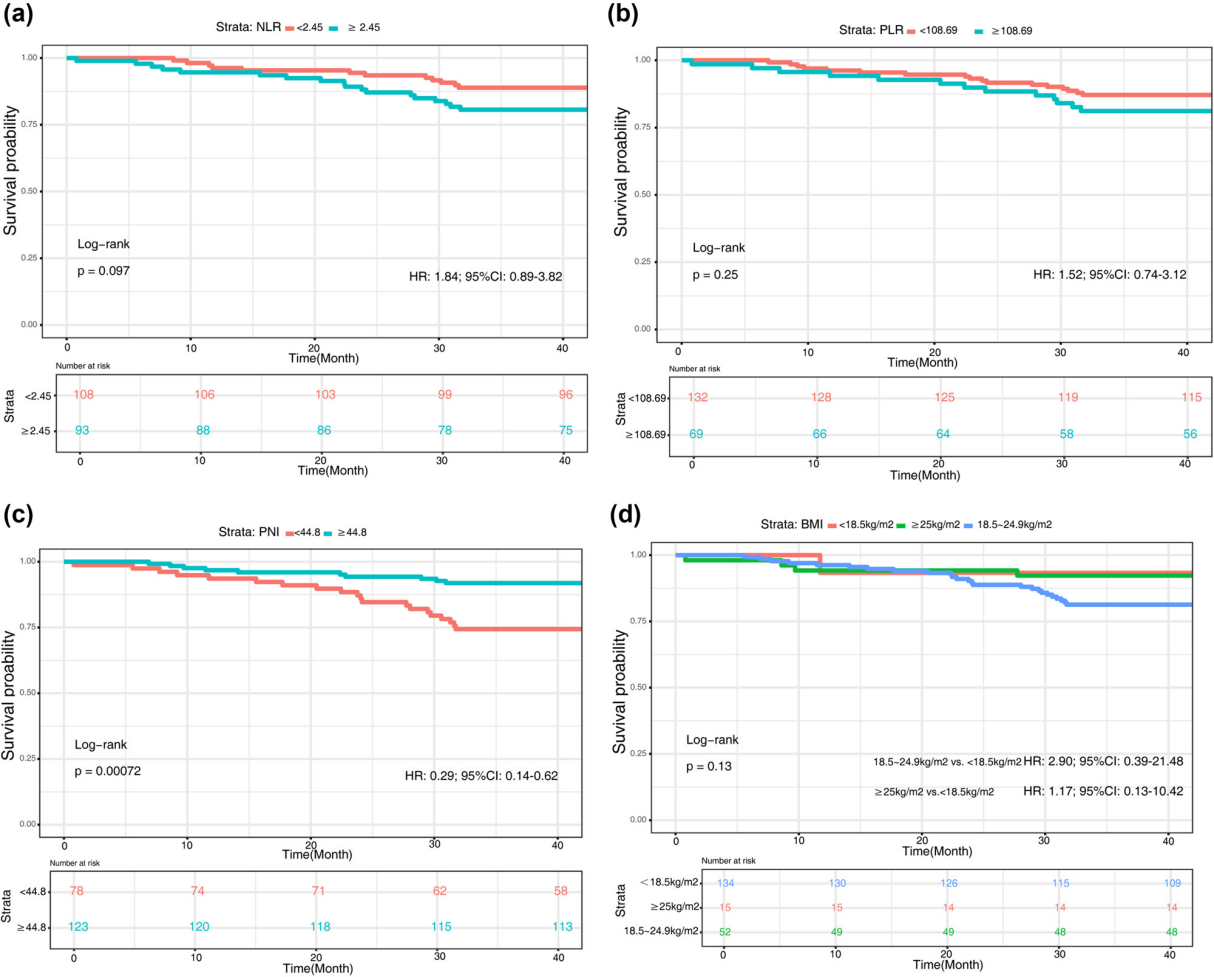
Kaplan–Meier curves showing the associations of the NLR, PLR, PNI, and BMI with OS. (a) NLR; (b) PLR; (c) PNI; (d) BMI.

The correlations between the NLR or PLR and OS are depicted in Figure 2a and b. The 1- and 3-year OS rates for patients with NLRs <2.445 were 104/108 (96.3%) and 96/108 (88.9%), respectively. For patients with an NLR ≥2.445, the 1- and 3-year OS rates were 88/93 (94.6%) and 75/93 (80.6%), respectively. Compared to patients with low NLRs, those with high NLRs showed no statistically significant difference in OS (*χ* ^2^ = 2.8, *P* value = 0.10). The 1- and 3-year OS rates for patients with a PLR of <180.69 were 127/132 (96.2%) and 115/132 (87.1%), respectively. The 1- and 3-year OS rates for patients with a PLR of ≥180.69 were 65/69 (94.2%) and 56/69 (81.2%), respectively. There was no significant difference in OS between patients with high PLRs and those with low PLRs (*χ* ^2^ = 1.3, *P* = 0.3). The 1- and 3-year OS rates for patients with PNI < 44.48 were 73/78 (93.6%) and 58/78 (74.4%), respectively. For patients with a PNI of ≥44.48, the 1- and 3-year OS rates were 119/123 (96.7%) and 113/123 (91.9%), respectively. Compared with patients with low PNI, those with high PNI had significantly better OS (*χ* ^2^ = 11.4, *P* = 0.001) (Figure 2c). Additionally, there was no significant difference in OS when comparing different BMI strata (*χ* ^2^ = 4, *P* = 0.1) (Figure 2d).

### 3.5 K‒M curves showing the associations of the NLR, PLR, PNI, and BMI with OS

**Figure 3:**
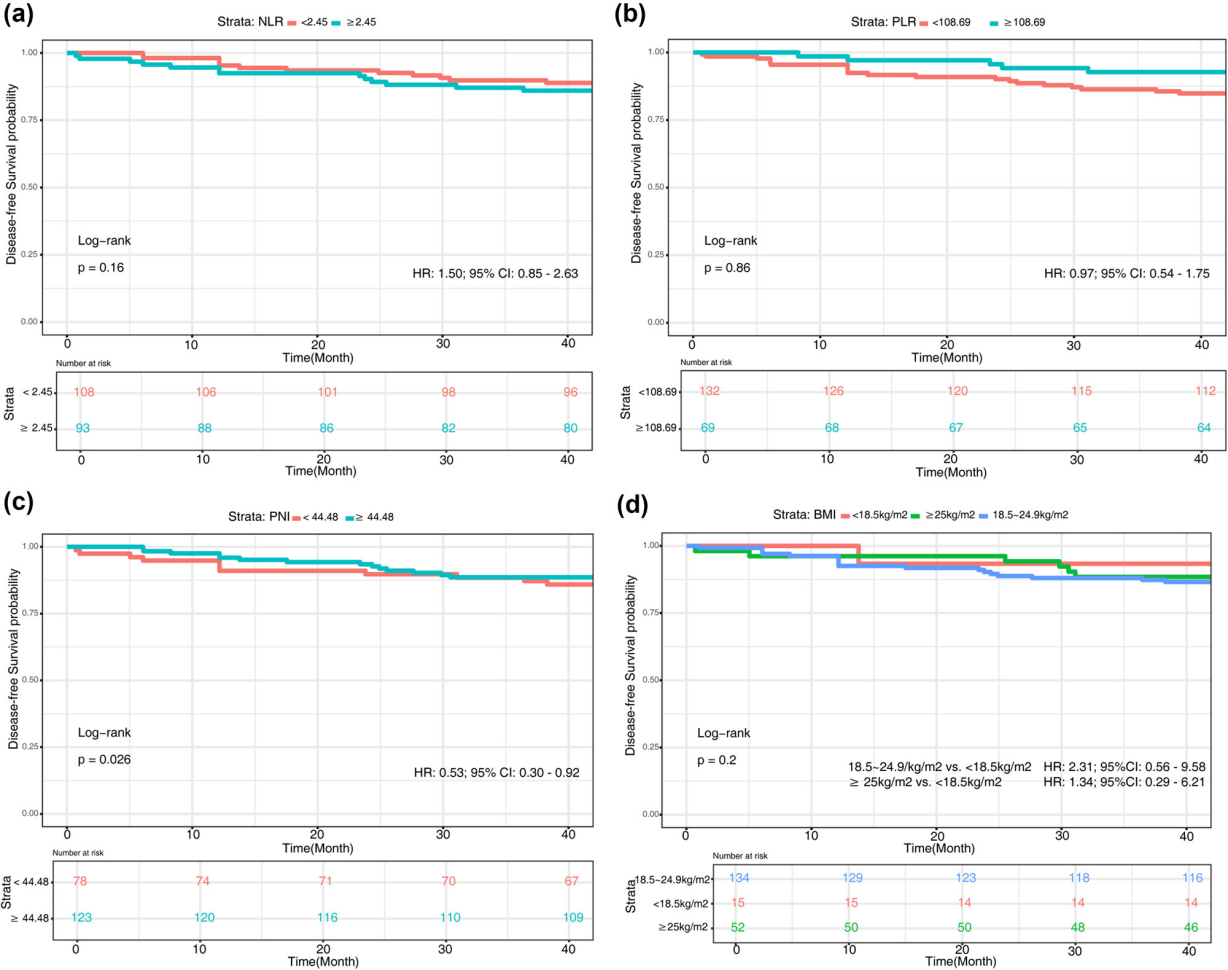
Kaplan–Meier curve of the associations of the NLR, PLR, PNI, and BMI with DFS. (a) NLR; (b) PLR; (c) PNI; (d) BMI.

For the DFS data, categorization continued to be based on the cutoff value derived from the OS data. No significant difference was observed between groups categorized by NLR (Figure 3a) or PLR (Figure 3b), either below or above their respective cutoff values. Among the PNI groups, those with a higher PNI exhibited better DFS (*χ* ^2^ = 4.9, *P* = 0.03) (Figure 3c). Furthermore, no significant difference was observed in DFS across different BMI strata (*χ* ^2^ = 3.2, *P* = 0.2) (Figure 3d).

### 3.6 The associations of the NLR, PLR, PNI, and BMI with OS according to Cox regression analysis

Univariate Cox regression analysis, with a PNI of <44.48 as the reference category, indicated that the group with a PNI of ≥44.48 had a more favorable survival outcome (*P* = 0.001). However, after adjusting for age, sex, tumor grade, TNM stage, tumor type, and the decision to administer chemotherapy, multivariate Cox regression analysis revealed no statistically significant differences between the PNI ≥ 44.48 and PNI < 44.48 groups (*P* = 0.538) (Table 7). Comprehensive results of the multivariate Cox regression analysis are presented in Table S1.

**Table 7:** Cox regression analysis of the associations between the NLR, PLR, PNI, and BMI and OS

Variables	Univariate analysis				Multivariate analysis^#^			
	HR	Lower 0.95	Upper 0.95	Pr(>|*z*|)	HR	Lower 0.95	Upper 0.95	Pr(>|*z*|)
NLR < 2.45	Ref.				Ref.			
NLR ≥ 2.45	1.839	0.886	3.819	0.102	2.250	0.941	5.378	0.068
PLR < 108.69	Ref.				Ref.			
PLR ≥ 108.69	1.517	0.737	3.124	0.258	1.407	0.597	3.314	0.435
PNI < 44.48	Ref.				Ref.			
PNI ≥ 44.48	0.292	0.137	0.624	0.001	0.873	0.360	2.115	0.763
BMI < 18.5 kg/m^2^	Ref.				Ref.			
18.5–24.9 kg/m^2^	2.910	0.394	21.475	0.295	16.01	0.610	419.810	0.096
≥25 kg/m^2^	1.165	0.130	10.420	0.892	22.535	0.379	1340.524	0.135

^#^Adjusted by age, sex, tumor difference, TNM stage, type of cancer, and chemotherapy.

## 4 Discussion

The inflammatory response and nutritional status are often related to the survival prognosis of tumor patients, and indicators such as the NLR, PLR, PNI, and BMI are low-cost to measure and identify and can be easily incorporated into routine clinical practice. This study aimed to analyze the prognostic value of the NLR, PLR, PNI, and BMI for survival in patients with colorectal cancer. The results showed that in patients with colorectal cancer, the PNI possesses a certain predictive value, and the predictive AUC for OS was 67.31%. In colon cancer, the predictive AUC was 71.98%. When the PNI is categorized based on the cutoff value, patients with a greater PNI exhibit greater survival benefits, as indicated by improved DFS and OS.

The role of host inflammation in influencing tumor growth and spread is complex and controversial. Tumor progression may result from an imbalance between protumor (inflammatory) and antitumor (immune) forces. Platelets facilitate cell adhesion and release growth factors that promote tumor growth and invasion. Immune cells, such as macrophages and lymphocytes, can induce tumor angiogenesis and extracellular matrix remodeling and enhance cell proliferation and metastasis through the activation of extracellular matrix degradation and the production of growth factors and proteases [22,23]. Lymphocytes are fundamental to both the innate and adaptive immune systems and play a critical role in immune surveillance and immune editing for sculpting the tumor microenvironment [24,25,26].

Lymphocytes can execute antitumor immune responses by enhancing tumor cell apoptosis and inhibiting tumor cell proliferation. Lymphocytes that infiltrate the tumor microenvironment act as initiators of antitumor immune responses. Reduced lymphocyte infiltration within the tumor microenvironment signifies an impairment in the host’s immune system and a diminished antitumor immune response. The formation of an environment with low lymphocyte infiltration is more conducive to the proliferation and metastasis of tumor cells [27,28]. Therefore, lymphopenia, along with a reduction in immune system function and defects in immune surveillance, can result in the activation of proto-oncogenes and the suppression of tumor suppressor genes, thereby contributing to tumorigenesis and the progression of tumor development [26,29]. In the tumor microenvironment, complex interactions between inflammatory cells and mediators can manifest in the peripheral blood circulation. These interactions are reflected in biomarkers; elevated NLR and PLR values are indicative of a protumor environment that favors tumor growth, invasion, and metastasis.

Currently, NLR, PLR, BMI, and related markers are frequently used to predict the prognosis of colorectal cancer patients, but clinical evidence remains inconsistent. For example, Chan et al. analyzed the prognostic value of NLR and PLR for colorectal cancer patients and reported no significant association with survival outcomes. The cut-off values for NLR and PLR in their study were 3.19 and 258, respectively, determined using an iterative testing method [30]. In contrast, Ming-Sheng et al. found that NLR and PLR were associated with OS, employing ROC curve analysis to determine optimal cutoff values, consistent with our methodology [31]. Kocak et al. investigated patients with metastatic colon cancer and identified negative delta PNI as an independent predictor of poor OS and progression-free survival (PFS). However, delta NLR and delta PLR did not predict survival outcomes in their cohort [32]. These findings align with our results, highlighting the influence of confounding factors on PNI and its association with OS. A meta-analysis of the postoperative rectal cancer population indicated that an elevated NLR and PLR were associated with poorer OS. Concurrently, a high NLR is also linked to a reduced DFS [33]. Another meta-analysis of patients who underwent postradical resection for rectal cancer also revealed that a high NLR was correlated with poor OS and DFS [34]. An earlier analysis also revealed a strong association between the NLR and poor OS in colorectal cancer patients [35]. However, in this study, no significant differences in OS or DFS were found among populations categorized by the NLR or PLR. Inflammatory markers are numerous, interrelated, and weakly correlated with systemic factor profiles. This suggests that the NLR and PLR may not comprehensively represent the host’s inflammatory status [36]. Additionally, the inflammatory state of the body can be influenced by factors such as surgery. Preoperative inflammatory markers have been found to have poor accurate predictive performance [37]. Consequently, the profile of inflammatory factors can also change over the course of treatment. Thus, varying measurement time points may also influence the outcomes.

As an effective indicator of immunonutrition status, the PNI was used to assess surgical nutritional status and predict surgical risks and complication rates. The PNI is calculated based on the serum albumin concentration and total lymphocyte count. In recent years, research has indicated that the PNI is associated with the prognosis of cancer patients [38,39,40]. BMI is another effective indicator used to assess the immune-nutritional status of patients. A low BMI (≤18.5 kg/m^2^) often indicates immunosuppression. Previous studies have linked low BMI to the risk of postoperative complications for gastrointestinal tumors [19,41]. However, an increasing body of evidence suggests that BMI is also associated with tumor prognosis [42,43,44].

In this study, the PNI demonstrated superior predictive accuracy for survival outcomes than did the NLR or PLR. Differences in survival outcomes were observed among patients categorized by PNI. In patients with advanced colorectal cancer and liver metastasis, a lower PNI is associated with poorer PFS and OS, and the PNI could be an independent prognostic factor for OS [21]. This suggests that nutritional status, as reflected by the PNI, is a significant host-related prognostic indicator [45]. In patients who underwent radical laparoscopic colectomy, a lower preoperative PNI was significantly associated with postoperative complications and a poor survival prognosis [46]. The current meta-analysis suggested that for the colorectal cancer population, a low PNI is significantly associated with poor OS, cancer-specific survival, and DFS are also associated with the incidence of postoperative complications [47]. Given that the tumors studied are within the digestive system, their local impact may more noticeably affect the patient’s nutritional status. For other digestive system cancers, a low PNI is also associated with poor OS and the occurrence of postoperative complications [48].

In addition, PNI was significant in univariate analysis but lost significance in multivariate analysis, likely due to insufficient adjustment for confounding variables. For instance, in the stratified analysis of PNI groups, treatment methods such as chemotherapy and radiotherapy were strongly correlated with PNI groups. In groups with higher PNI, a notably higher proportion of individuals received chemoradiotherapy. Additionally, PNI was notably correlated with age groups. These findings suggest that PNI is influenced by the above potential factors, which may impact its observed relationship with OS. Guo et al. investigated the role of neoadjuvant and adjuvant immunotherapy in colorectal cancer. Their comprehensive study summarized the utility of neoadjuvant chemotherapy in improving the tumor microenvironment and cancer immunology while exploring its intricate associations with tumor biomarkers and microsatellite instability status [49]. This elucidates the confounding factors in our multivariate regression, particularly the observed positive association between PNI and chemoradiotherapy. Moreover, PNI indirectly reflects the body’s chronic inflammatory state, wherein albumin and lymphocyte counts are gradually depleted. Guo et al. also highlighted the pivotal role of Nrf2 in regulating tumor oxidative stress, a key mechanism driving chronic inflammation [50]. The inhibition of oxidative stress through Nrf2 modulation may improve chronic inflammation and subsequently elevate PNI levels, potentially enhancing patient prognosis.

The precision medicine revolution has had a transformative impact on the treatment of advanced cancers. However, colorectal cancer, one of the leading cancer-related causes of death, has seen comparatively slower advancements [51]. Future studies should prioritize exploring combined molecular markers and dynamic monitoring of marker changes, as this represents a promising avenue of research. Colorectal cancer is a heterogeneous disease marked by numerous molecular alterations that drive the dysregulation of signaling pathways, leading to tumorigenesis, progression, and invasiveness. Three major pathways have been identified in colorectal cancer pathogenesis: chromosomal instability, high microsatellite instability (MSI-H), and CpG island methylation phenotype. Given the heterogeneity of colorectal cancer, integrating multiple molecular markers with machine learning models could significantly enhance prognostic accuracy [52,53]. Dynamic biomarker changes offer valuable insights into tumor characteristics. For instance, fluctuations in CEA levels are predictive of treatment response and OS outcomes in colorectal cancer patients [54]. Furthermore, abnormal methylation of tumor suppressor genes and retrotransposon hypomethylation, detectable in circulating free DNA (cfDNA), suggest that dynamic methylation changes may serve as both diagnostic and prognostic markers [55]. Future studies integrating combined molecular markers, dynamic detection methods, and artificial intelligence models could improve survival outcome predictions with greater precision.

The limitations of this study include the following: first, the small sample size of this study diminishes the robustness and reliability of the findings. Additionally, insufficient data limited our ability to perform subgroup analyses for different population types, particularly for detailed exploration of populations with varying tumor sites, to derive more tailored conclusions. Future studies should address these limitations by increasing the sample size and conducting multicenter research to validate the conclusions. Second, the analysis in this study did not encompass the full spectrum of inflammatory cells and factors, thus limiting our ability to draw a more comprehensive conclusion regarding the relationship between inflammation and the prognosis of patients with colorectal cancer. Therefore, future research should aim to identify a broader and more innovative set of inflammatory and nutritional indicators, and multicenter, large-sample studies should be undertaken to confirm the associations between inflammation, nutritional status, and survival prognosis in colorectal cancer patients.

## Supplementary Material

Supplementary Table
