# Supplementary material for: The prognostic value of the neutrophil-to-lymphocyte ratio, platelet-to-lymphocyte ratio, and prognostic nutritional index for survival in patients with colorectal cancer
Source: Open Med (Wars). 2025 Jun 11;20(1):20251214. doi: 10.1515/med-2025-1214 (PMC12163575; doi:10.1515/med-2025-1214)
Supplement: Supplementary Table [file med-2025-1214-sm.pdf]

Supplementary material

Table S1: Comprehensive results of the multivariate COX regression analysis based on OS outcome

Variables	HR	lower 0.95	upper 0.95	Pr(> |z|)
NLR(≥2.45 vs <2.45)	2.25	0.94	5.38	0.068
Sex (Female vs Male)	0.62	0.25	1.54	0.303
Age (Continuous)	1.05	1.01	1.1	0.010
BMI (Continuous)	1.01	0.91	1.11	0.881
Differentiation (Continuous)	0.33	0.17	0.63	0.001
T(Continuous)	1.24	0.61	2.54	0.548
N (1 vs 0)	3.75	2.14	6.55	<0.001
Tumor type (Rectal vs colon)	1.15	0.49	2.72	0.750
Tumor type (Colorectal vs colon)	2.97	0.33	26.58	0.331
Chemotherapy (Yes vs No)	0.33	0.12	0.87	0.025
Radiotherapy (Yes vs No)	0.04	0.01	0.33	0.003
PLR (≥108.69 vs <108.69)	1.41	0.6	3.31	0.435
Sex (Female vs Male)	0.56	0.22	1.39	0.21
Age (Continuous)	1.06	1.02	1.11	0.002
BMI (Continuous)	1.01	0.93	1.11	0.757
Differentiation (Continuous)	0.42	0.23	0.77	0.005
T(Continuous)	1.14	0.57	2.26	0.71
N (1 vs 0)	3.45	1.97	6.03	<0.001
Tumor type (Rectal vs colon)	1.25	0.49	3.22	0.639
Tumor type (Colorectal vs colon)	3.19	0.35	28.78	0.302
Chemotherapy (Yes vs No)	0.37	0.14	0.94	0.037
Radiotherapy (Yes vs No)	0.05	0.01	0.37	0.004
PNI (≥44.48 vs <44.48)	0.87	0.36	2.11	0.763
Sex (Female vs Male)	0.54	0.22	1.35	0.187
Age (Continuous)	1.06	1.02	1.11	0.008
BMI (Continuous)	1.01	0.93	1.11	0.779
Differentiation (Continuous)	0.42	0.23	0.76	0.004
T(Continuous)	1.16	0.58	2.33	0.674
N (1 vs 0)	3.54	2.01	6.21	<0.001
Tumor type (Rectal vs colon)	1.1	0.45	2.65	0.835
Tumor type (Colorectal vs colon)	3.22	0.36	29.2	0.299
Chemotherapy (Yes vs No)	0.35	0.14	0.9	0.03
Radiotherapy (Yes vs No)	0.05	0.01	0.41	0.005

(*Continued*)

Table S1: *Continued*

Variables	HR	lower 0.95	upper 0.95	Pr(> |z|)
BMI (18.5–24.9 vs <18.5)	16.01	0.61	419.81	0.096
BMI (≥25 vs <18.5)	22.53	0.38	1340.52	0.135
Sex (Female vs Male)	0.43	0.17	1.10	0.079
Age (Continuous)	1.05	1.01	1.10	0.013
BMI (Continuous)	0.9	0.76	1.06	0.217
Differentiation (Continuous)	0.4	0.22	0.74	0.004
T (Continuous)	1.29	0.6	2.75	0.517
N (1 vs 0)	3.93	2.22	6.97	<0.001
Tumor type (Rectal vs colon)	0.95	0.38	2.34	0.909
Tumor type (Colorectal vs colon)	3.29	0.38	28.69	0.282
Chemotherapy (Yes vs No)	0.25	0.09	0.69	0.007
Radiotherapy (Yes vs No)	0.05	0.01	0.39	0.004
